# Retrospective Observations on the Ability to Diagnose and Manage Patients with Asthma through the Use of Impulse Oscillometry: Comparison with Spirometry and Overview of the Literature

**DOI:** 10.1155/2014/376890

**Published:** 2014-02-09

**Authors:** Constantine Saadeh, Blake Cross, Charles Saadeh, Michael Gaylor

**Affiliations:** ^1^Amarillo Center for Clinical Research (ACCR), TX, USA; ^2^Allergy, Asthma, Rheumatology Treatment Specialist (Allergy ARTS), 6842 Plum Creek Dr, Amarillo, TX 79124, USA; ^3^Texas Tech University Health Sciences Center, USA; ^4^University of North Texas School for Osteopathic Medicine, USA

## Abstract

*Objective*. Impulse oscillometry (IOS) is an evolving technology for the diagnosis and followup of patients with asthma. Our objective is to review the findings on patients who underwent both spirometry and IOS during clinical evaluations of their asthma. The goal was to retrospectively evaluate IOS during the initial diagnosis and followup of patients with asthma in comparison with spirometry. *Methods*. We routinely perform IOS and spirometry evaluation in patients with suspected asthma during baseline visits and at followup. We reviewed the data on 39 patients over the age of 13 with asthma at baseline and following treatment with inhaled corticosteroids. IOS and spirometry were both done at baseline, following short acting bronchodilator administration, and at followup after at least three months of inhaled corticosteroid treatment. *Results*. IOS showed improvement in airway function both initially, following short acting bronchodilator introduction, and later after initiation of long term inhaled corticosteroid treatment, even when the spirometry did not reveal improvement. We noted the IOS improvement in the reactance or AX as well as the resistance in smaller airways or R5. *Conclusion*. IOS may provide a useful measure towards identifying an asthma diagnosis and followup without inducing the extra respiratory effort spirometry requires.

## 1. Introduction and Background 

Impulse oscillometry measures both small and large airways resistance and resonance capacitance of the lung [1]. Its main advantage is its ability to perform these measurements in a noninvasive, relatively effort independent, and minimally intrusive manner during spontaneous normal tidal breathing [1–3].

In contrast to traditional spirometry, impulse oscillometry or IOS traces its findings independent of age, height, weight or gender on adolescents and adults aged 13 years or older [1, 4]. The most relevant outcome of IOS measures include R5 (resistance in small airways), R15 or higher (resistance in larger airways), and AX (low frequency integrated impedance reactance at R5).

These values can be compared to baseline following short acting bronchodilator use or longitudinally while patients are under treatment for chronic asthma via inhaled corticosteroids [5, 6].

IOS has been applied in few studies in asthma diagnosis and management. In these studies [4, 5, 7–13] asthma had already been diagnosed, and patients were included based upon symptoms and baseline abnormal spirometry. In this report, we reviewed the data on patients with initial symptoms of allergic asthma and allergic rhinitis in our clinic who had spirometry and IOS at baseline, following bronchodilator administration, and later, after treatment with inhaled corticosteroids. In this report the role of IOS utilization in routine clinical practice is examined retrospectively at both baseline and followup.

## 2. Methods

- 39 patients aged 13 years and older with a history of asthma or unexplained shortness of breath with allergic rhinitis were routinely evaluated at baseline with spirometry and IOS. The diagnosis of asthma was made by history of wheezing, cough, or shortness of breath. Some of the patients also reported history of asthma prior to coming to this office for evaluation. However, they have not been treated for asthma before. Family history of asthma was also confirmed in at least 30 of these patients. Patients with smoking history have been excluded. Also, by reviewing the charts as well patients with history of secondhand smoke were also excluded. The patients were routinely given a nebulized bronchodilator and the same measurements were obtained following its administration. Patients were then followed up at a minimum of three months of treatment regimens with various inhaled corticosteroids. The same measurements were obtained and recorded. IRB approval was granted for this study.
- Spirometry and IOS diagnostics were conducted utilizing a Jaeger (c) instrument. The technique of IOS measurement was as described in [1, 14]. Briefly patients were seated comfortably in a nonswivel chair. Nose clips were applied, and a special mouthpiece was used. Patients were allowed to breathe normally while a loudspeaker component of the instrument delivered intermittent multifrequency impulses over a minimum of 30 second duration. A trained technician guided, comforted, and assisted the patient in following the tracing as at least 3 sinusoidal readings were obtained. We chose the recording with the best coherence at frequencies from 5 to 30 Hz. The ideal coherence was 0.9, 1, 1, and 1 at 5, 10, 15, and 20 Hz, respectively. The technician was also trained to capture subclinical leaks through the mouthpiece and leak recordings were discarded. The values we obtained were recorded as R5, R15, and AX (the integrated impedance reactance at R5 and above). We then recorded spirometry after IOS in the same setting. Forced expiratory volume of the first second, or FEV1, was recorded and the results were obtained according to the guidelines of the American Thoracic Society.
- We tabulated results as outlined below. Interference from cough, swallowing, or breath holding was identified and discarded during spirometry. Patients were treated with various inhaled corticosteroids (ICS) or ICS/LABA (inhaled corticosteroid and long acting beta agonist combination). We noted posttreatment results for at least three months later. We gathered histories and conducted exams also. All patients had reported symptoms and a history of asthma with or without allergic rhinitis.
- Statistics. Data were analyzed via the (Welch's) *t*-test utilizing the *t*-test comparison. A statistically significant difference was considered at *P* value of less than or equal to 0.05.

## 3. Results 

**Figure 1:**
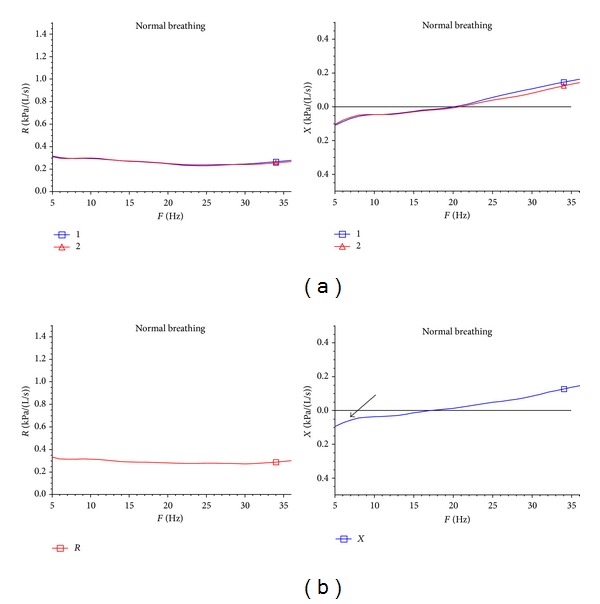
(a) Patient 15 before bronchodilator. (b) Patient 15 after bronchdilator.

**Figure 2:**
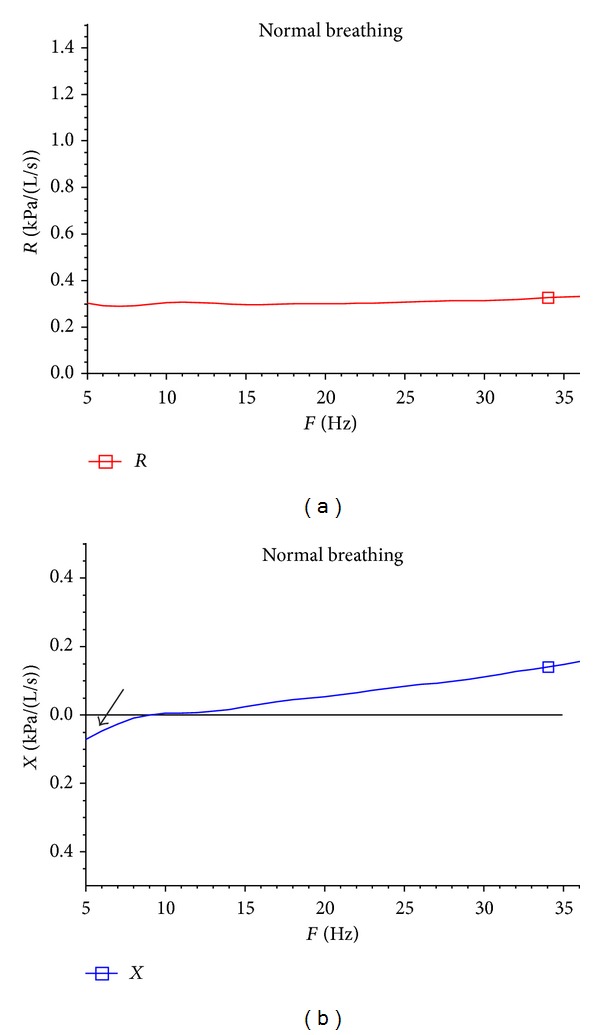
Patient 15 followup (post) (shadowed area AX).

Table 1 summarizes the demographic and background information of the patients as well as the results of pre- and post-ronchodilator and Table 2 summarizes the follow-up treatment of at least three months. In both situations the IOS, and particularly the AX, provided a reliable indicator of improvement. FEV1 did show improvement in some patients, but AX consistently showed improvement in almost every patient followed after consistent inhaled corticosteroid use.  Figure 1 illustrates a typical patient with asthma and the IOS measurement before and after administration of a short acting Beta agonist (levalbuterol) at baseline evaluation. Also in Figure 1, we included in the third column the type of inhaled corticosteroids, alone or in combination, that the patients were placed on at presentation.  Figure 2 shows the same patient following treatment with inhaled corticosteroids and improvement in AX. The retrospective clinical data we utilized and present appear to support the hypothesis that IOS may play an important role in evaluating and following patients with asthma—even when the baseline FEV1 is normal or does not change with treatment.

FEF 25–75% was also measured in the same patients. Improvement from a low abnormal (<80% level of predicted) was noted in only five patients. Improvement from a normal baseline of ≥80% of predicted was noted in nine patients. The improvement was defined as a change of 15% of predicted over baseline after nebulizer from baseline or after inhaled corticosteroid treatment.

Based on our data, even though the FEF 25–75% was helpful in few patients, the majority of patients did not show a response pattern for FEF 25–75% (data not shown).

## 4. Discussion

In adults aged 13 years and older, the R5 value in cm of H_2_O is the summation of large and small airways [1, 15, 16]. Typically R5 is approximately 3 cm H_2_O or less. R15 is a direct measure of larger airways and R15 is about 2 cm H_2_O or less. Therefore, absolute measurement of small airways is R5–R15 [1, 15, 16]. R5 in general is the larger number than R15 or R20. Therefore, R5 is a reflection of small airways while R15 is directly correlated with larger airways. It is possible that R5 can be equal to R15 or R20 and the difference, therefore, will be 0. In this situation, it only reflects that the airways are completely normal [1].

AX by definition is the area under *X* curve and is reflective of the reactance of the lung in response to the instrument's loudspeaker stimuli. This value reflects the integral reactance of small airways, and may by itself be an index of small airway response to the external application of multiple frequency signals through a transducer. X5 or resonant frequency is the point on the curve reflective of the same reactance as AX or at 5 Hz. Since AX is reflective of physiologic integration of small frequency signals rather than a specific point in the respiratory cycle, we elected to choose AX to reflect the lung reactance rather than X5. This was our reasoning. However, we realize that some studies have preferred to include X5 rather than AX. The literature is still not clear in this regard at this point [1, 15]. We do realize that we have chosen the average of inspiration and expiration AX. In our data, we chose patients with clear asthma that have almost equal AX during inspiration and expiration. In our unpublished observations, we have noted that in some patients particularly with COPD, the AX on expiration is at least twice that of inspiration. It is our belief that this is related to vocal cord dysfunction. We therefore have excluded patients with major differences between AX on inspiration and expiration.

The concept of forced oscillation technique or FOT was initiated in 1956 by Dubois [1]. Later, Lancer in 1976 introduced it as a resonant frequency between 6 and 11 Hz [1]. However, measurements of resistive frequencies at 4 to 32 Hz were noted to comprise small airway resistance [1]. In 2003 we presented data on IOS responses including decrease in R5 and AX, even in patients who had normal spirometry, including patients who had normal spirometry via the FEV1 or those who have decreased initial FEV1 [2].

These patients had an FEV1 with minimal to no improvement with inhaled corticosteroids [17]. Yet, their impulse oscillometry improved significantly. The IOS is a modification of FOT whereby the IOS delivers a regular square wave of pressure five times per second [1]. This has the advantage of generating a larger sample during measurements and omitting a continuous spectrum of frequencies (5–35 Hz) that provide a more detailed characterization of respiratory function [1, 6]. IOS therefore measures the properties of the lung to an externally applied stimulus. This is achieved through applying pressure variations at the mouth of the subject via a loudspeaker component of the instrument. Respiratory impedance is then obtained as resistance (R5 and above) and reactance (AX).

In our clinic, we utilize IOS routinely to determine the status. In this study, we chose our patients at random and as part of their asthma evaluation and management. These patients presented with allergic rhinitis and history of shortness of breath. Some of them were told that they had asthma by the referring provider. These patients were only treated by the referring provider with as needed short acting beta agonist inhaler only.

We realize the limitations of this study. First, this is retrospective evaluation. Ideally, prospective evaluation is more appropriate to study the effects of IOS in the diagnosis of asthma. However, since we perform IOS routinely and have well trained technicians, we thought that the retrospective data presentation may give awareness of the technical use of this modality in studying the pulmonary status of patients suspected to have asthma.

Second, there were three patients in our cohort that showed decrease or no reversibility in the FEV1 following bronchodilator treatment as shown in Table 1. These were patients 1, 2, and 18. Patients 1 and 18 had decrease in the FEV1 by 19 and 11%, respectively. Patient 2 did not show much change in FEV1 following hand-held nebulizer treatment. This is not unusual in clinical practice perhaps because of either poor cooperation or fatigue factor. It is important to note, however, that, even though the FEV1 decreased, in all of these three patients the IOS values significantly improved. This might suggest that support effort effect may play a role even though the spirometry tracing appeared to be appropriate. For this reason, it is reasonable to perform IOS since it is effort independent prior to spirometry to get more accurate readings. In our experience, performing spirometry prior to impulse oscillometry can lead to erroneous elevation in the AX. This is perhaps due to the provoking of the lung mechanics during spirometry (unpublished observations).

Third, the followup of these patients was a minimum of three months to a maximum of 18 months. In this case, the FEV1 may decrease with age but only slightly within a period of 18 months. The IOS values, however, should not change. In patients with severe obesity, there might be a minor effect on impulse oscillometry in terms of elevating the value of X5 or AX [18]. In reviewing the status of our patients based on what has been reported in the literature, there should not a significant effect on the values of the impulse oscillometry.

Fourth, our patients represent a heterogeneous group since they were placed on different types of inhaled corticosteroids. However, they were all evaluated by the same short acting beta agonist nebulizer which was levalbuterol hydrochloride. We could have chosen albuterol but based on the literature the effects are similar. However, irrespective of which kind of corticosteroid was used, the improvement in IOS appeared to be uniform.

Despite these limitations, we may have been able to demonstrate that IOS is useful in the diagnosis and followup of patients with adult asthma. Previously, IOS has been regarded as equivalent but not as an alternative to spirometry [4, 6]. Marotta et al. showed that, for children at risk for asthma, IOS is a better predictor than spirometry [16]. In a study by Al-Mutairi et al. [19] patients with COPD and asthma diagnoses were tested via IOS and spirometry. The authors concluded that IOS may be an alternative method to evaluate lung function at baseline when compared to spirometry.

Our observations, however, were directed towards evaluating patients at baseline following bronchodilator administration and at followup with ICS or ICS/LABA. These observations were retrospective and involved a relatively small number of adult asthma patients. It is noteworthy, however, that longitudinal studies in adult asthma with IOS evaluation should be considered in a larger cohort of patients.

In children, IOS has been more evaluated, even prospectively. Ortiz and Menendez showed that salmeterol alone showed IOS improvement in children between the ages of 2 and 5 when spirometry could not be performed in this age group [20]. In another larger cohort study, Komarow et al. demonstrated the efficacy of IOS in 117 children even as an alternative to FEV1 in asthmatic children [21].

In a study by Schermer et al. [22], IOS was studied in metropolitan firefighters. Similar to our findings, R5 and X5 (equivalent to AX; see earlier) were better predictors of airway dysfunction, even when the spirometry was normal in most of these subjects. In airway dynamics IOS offers an advantage in measuring bronchomotor tone in adolescents who have asthma with daily variation, even when spirometry was unchanged (Goldman and Carter) [23]. In another study by Meraz et al. [13], IOS indices were sensitive indicators of small and large airway resistance in patients with asthma and cystic fibrosis independent of the upper airway shunt capacitance.

Other investigators have shown that X5 can be useful parameter of bronchial hyperresponsiveness in children [24]. X5 was suggested to be a useful correlate and adjunct to nitric oxide measurement in COPD and asthma [25]. In reality X5 referred to as resonant frequency is a direct reflection of AX [1, 6]. FEF 25–75% is obtained during routine pulmonary function testing. It measures the air flow during the mid-expiration cycle. It is supposed to be reflective of small airway resistance. In reality, however, it is a variable parameter and is also dependent on comparative parameters such as age, height, weight, and gender [26]. In our data the FEF 25–75% was helpful in a minority of patients (total of fourteen patients). In a study by Drewek et al., the FEF 25–75% was noted to be a valuable parameter to measure small airway decline in methacholine responsiveness in children [27]. Alberts et al. demonstrated that FEF 25/75 is useful in the diagnosis of asthma when the cut-off value at baseline is less than 60% [28]. Rao et al. showed that FEF 25/75 can be a predictor of childhood asthma and severity when the FEV1 is normal [29]. These studies did not utilize IOS in their evaluation of small airway disease. In a recent study by Anderson et al., baseline values of FEV1 in patients with persistent asthma according to the British Thoracic Society asthma treatment steps did not differ after inhaled corticosteroids while the R5 did show improvement [30]. AX as utilized in our study was not noted in this study. In another study, Yamaguchi et al. examined IOS and specifically AX in patients with asthma and noted improvement in AX from baseline when using HFA-BDP (Hydrofluoroalkane-beclomethasone dipropionate) compared to chlorofluorocarbon-beclomethasone dipropionate (or CFC-BDP). In their study, there was no comparison to spirometry, but they did affirm the role of IOS in the evaluation of small airway disease [31]. In our study and with our limited observations, the role of IOS and especially AX has been noted to be clinically significant. It did not correlate with FEV1 or FEF 25/75. This observation is important and warrants further investigation in a prospective large scale trial analysis.

Additional applications of IOS which we applied in our clinic and others as well include response to exercise in patients with symptoms suggestive of asthma who have normal spirometry and AX [32]. Also, it has been utilized in methacholine challenge testing [11] and in pregnancy when patients could not perform spirometry [33].

As previously stated, AX is easier to observe since it is reflective of the integral of reactance at various frequencies, particularly the small airways. We relied on R5 and AX in our study to evaluate these patients. In order to account for reliability and accuracy of IOS testing, the concept of coherence was used. Coherence is the estimate of the quality of impedance measurements. This approach provides an index of discrepancy between input and measure signals. The appropriate coherence established at each frequency of resistance is as follows: Co5 is 0.9, Co10 is 1.0, Co15 is 1.0, and Co20 is 1.0 (R15 is considered a measurement of larger airway resistance) [1, 6]. Based on the above, interpretation of the IOS requires more training and experience on the part of both the technician and interpreting physician. The technician should be alert to the patient's breathing, mouthpiece position, tidal breathing, and selection of the best normal breathing wave in relationship to coherence and absence of leak through the mouthpiece. The physician interpreter should have the basic knowledge of the pathophysiology of IOS and the ability to detect aberrancies in AX or R5 [1, 6].

Some of the pitfalls of IOS include airway leak and poor holding of the cheeks (which is particularly important in children and COPD patients). Tongue effect, cough, swallowing, shallow breaths, and vocalization are other pitfalls [1]. An experienced technician is able to identify these pitfalls and perform an appropriate test. Our technicians were well trained in identifying these pitfalls.

Other uses and future utilizations of IOS include respiratory impedance model measurements [34], heart failure models, and ventilatory changes following head-up tilt standing in healthy subjects [35–37].

Despite the above limitations, IOS has been shown to be useful in interpreting small airway dysfunction and, perhaps, superior to the FEF 25–75 [38]. FEF 25–75 measurement, because of its dependence on effort, can lead to false positive findings since its effect can diminish with time [30].

## 5. Conclusion

In our small observational study, we noted retrospectively that when IOS is performed appropriately it can potentially be an additional and perhaps be considered an alternative tool in the diagnosis and followup of adult asthma patients. The limitation of our study being retrospective and heterogeneous undermines a firmer conclusion. Future studies in a prospective group of of patients with adult asthma should help define a clearer role of IOS utility in asthma diagnosis and followup.

## Figures and Tables

**Table 1:** Patient Spirometry and IOS Baseline.

Asthma patient	Age	Inhaled steroid	Before FEV1	Post bronchodilator FEV1	% Chg	Before AX	Post bronchodilator AX	% Chg	Before R5	Post bronchodilator R5	% Chg	Before R15	After R15	% Chg
1	41	Mometasone furoate	4.03	3.27	−19.00%	11.98	6.94	−42.10%	4.12	3.41	−17.30%	3.2	2.62	−18.30%
2	41	Fluticasone/salmeterol	4.16	4.12	−1.00%	10.37	6.36	−38.70%	4.03	3.86	−4.10%	2.83	2.77	−1.90%
3	45	Ciclesonide	3.6	2.94	−18.30%	4.44	5.24	17.90%	2.78	2.47	−10.90%	1.99	1.64	−17.50%
4	78	Mometasone furoate	2.26	2.74	21.60%	7.05	3.13	−55.50%	2.79	72.2	−15.90%	1.87	1.74	−7.20%
5	65	Budesonide/formoterol	1.5	1.79	19.10%	6.22	3.81	−38.70%	3.02	2.36	−21.80%	2.07	1.69	−18.20%
6	71	Beclomethasone dipropionate	1.37	1.28	−6.40%	32.15	24.39	−24.20%	6.18	5.25	−15.00%	3.74	3.71	−1.10%
7	44	Budesonide/formoterol	3.54	3.87	9.20%	11.82	2.49	−78.90%	4.55	3.21	−29.40%	3.85	2.91	−24.40%
8	47	Mometasone furoate	2.18	2.65	21.40%	7.74	5.59	−27.80%	3.62	3.48	−3.90%	2.53	2.62	3.70%
9	59	Ciclesonide	2.26	2.26	0.20%	8.71	6.75	−22.50%	3.71	3.24	−12.60%	2.49	2.21	−11.00%
10	35	Mometasone furoate	3.81	4.01	5.10%	7.4	6.5	−12.20%	3.01	3.56	18.20%	2.32	2.46	5.80%
11	60	Mometasone furoate	1.91	1.95	2.40%	23.86	19.05	−20.20%	6.47	5.29	−18.20%	3.98	3.31	−16.80%
12	31	Ciclesonide	4.13	4.13	−0.10%	2.28	1.04	−54.60%	2.72	2.43	−10.60%	2.29	2.29	−0.30%
13	51	Budesonide/formoterol	1.97	1.85	−6.00%	14.32	11.78	−17.70%	4.64	4.14	−11.00%	2.72	2.64	−2.80%
14	29	Mometasone furoate	3.93	4.18	5.30%	4.08	2.99	−26.70%	2.39	2.45	2.60%	1.92	1.8	−6.20%
15*	36	Mometasone furoate	3.61	3.89	7.80%	6.32	4.59	−27.30%	3.14	3.37	7.20%	2.76	2.94	6.70%
16	51	Budesonide	2.32	2.36	1.90%	7.43	5.63	−24.30%	2.79	3.11	11.50%	2.29	2.39	4.20%
17	50	Budesonide	3.11	3.25	4.70%	3.05	1.88	−38.20%	3.63	3.05	−15.90%	3.19	2.67	−16.30%
18	83	Fluticasone/salmeterol	2.49	2.21	−11.30%	6.36	9.18	44.30%	2.25	2.66	18.20%	1.84	1.8	−2.20%
19	57	Fluticasone/salmeterol	1.91	1.94	1.50%	22.44	20.31	−9.50%	4.3	4.18	−2.80%	2.75	2.67	−3.20%
20	37	Mometasone furoate	3.78	3.51	−7.10%	7.41	5.34	−28.00%	3.69	3.37	−8.60%	2.85	2.66	−6.90%
21	51	Ciclesonide	2.64	2.73	3.50%	9.4	7.54	−19.80%	4.01	3.31	−17.40%	2.88	2.48	−14.00%
22	24	Mometasone furoate	3.95	3.72	−5.70%	5.55	2.95	−46.80%	2.92	2.41	−17.40%	2.17	1.91	−12.00%
23	35	Budesonide/formoterol	2.38	2.86	20.50%	6.23	2.35	−62.20%	3.56	2.18	−38.70%	3.15	1.95	−38.20%
24	61	Budesonide/formoterol	2.48	2.56	3.20%	10.87	9.11	−16.20%	3.86	3.58	−7.30%	2.62	2.43	−7.30%
25	33	Mometasone furoate	2.74	2.73	−0.30%	9.86	9.56	−3.00%	3.66	4.09	11.80%	2.41	2.49	3.30%
26	49	Mometasone furoate	2.96	2.99	1.20%	7.66	6.06	−20.80%	3.97	3.86	−2.90%	3.06	3.05	−0.10%
27	65	Fluticasone/salmeterol	1.73	1.87	7.90%	4.78	5.55	16.10%	2.97	3.99	34.20%	2.4	3.66	52.50%
28	31	Mometasone furoate	2.56	2.19	−14.40%	5.37	2.96	−44.90%	3.73	3.38	−9.40%	2.64	2.8	6.00%
29	74	Mometasone furoate	1.6	1.67	4.90%	14.06	11.15	−20.70%	3.65	3.23	−11.50%	2.54	2.38	−6.30%
30	54	Ciclesonide	2.36	2.7	14.10%	5.23	2.08	−60.20%	2.6	2.18	−15.90%	2.05	1.87	−8.80%
31	54	Ciclesonide	2.53	2.54	0.40%	9.46	7.24	−23.50%	2.85	2.76	−3.10%	1.94	1.87	−3.60%
32	53	Ciclesonide	3.01	3.1	3.10%	20.7	18.9	−8.70%	4.83	4.97	2.80%	3.28	3.17	−3.30%
33	60	Budesonide/formoterol	1.63	1.7	4.40%	15.32	4.46	−70.90%	4.91	3.83	−22.20%	3.9	3.34	−14.20%
34	80	Mometasone furoate	2.3	2.54	10.10%	19.97	5.98	−70.00%	4.2	2.97	−29.40%	2.85	2.29	−19.80%
35	65	Fluticasone/salmeterol	1.69	1.8	6.70%	16.32	7.38	−54.80%	4.45	3.41	−23.20%	2.95	2.47	−16.30%
36	71	Mometasone furoate	1.56	1.67	6.70%	5.68	7.31	28.80%	2.11	3.08	46.20%	1.7	2.42	42.20%
37	23	Fluticasone/salmeterol	3.36	3.74	11.30%	21.19	5.63	−73.40%	4.39	2.85	−35.00%	2.86	2.38	−16.90%
38	63	Beclomethasone dipropionate	2.73	2.77	1.60%	6.05	4.69	−22.40%	3.07	2.83	−8.00%	2.33	2.22	−4.90%
39	52	Budesonide	1.81	1.97	8.60%	15.74	6.46	−58.90%	3.8	2.66	−30.00%	2.4	1.72	28.20%

*denotes patient that is referenced in Figure 1.

**Table 2:** Patient followup.

Asthma patient	Followup FEV1	Followup AX	Followup R5	Followup R15	FEV1 % chg	AX % chg	R5 % chg	R15 % chg
1	4.01	9.69	3.51	2.67	−0.50%	−19.12%	−14.81%	−16.56%
2	4.33	6.9	3.86	2.72	4.09%	−33.46%	−4.22%	−3.89%
3	3.25	7.87	2.88	1.85	−9.72%	77.25%	3.60%	−7.04%
4	2.57	4.03	2.5	1.84	13.72%	−42.84%	−10.39%	−1.60%
5	1.78	7.46	2.32	1.59	18.67%	19.94%	−23.18%	−23.19%
6	1.24	26.32	5.59	3.81	−9.49%	−18.13%	−9.55%	1.87%
7	3.42	7.82	3.74	2.97	−3.39%	−33.84%	−17.80%	−22.86%
8	3.44	7.77	3.14	2.12	57.80%	0.39%	−13.26%	−16.21%
9	—	6.81	3.05	2.14	—	−21.81%	−17.79%	−14.06%
10	4.08	4.94	2.62	2.06	7.09%	−33.24%	−12.96%	−11.21%
11	2.29	17.6	5.49	3.82	19.90%	−26.24%	−15.15%	−4.02%
12	3.68	1.3	2.19	1.89	−10.90%	−42.98%	−19.49%	−17.47%
13	2.01	8.58	3.61	2.28	2.03%	−40.08%	−22.20%	−16.18%
14	3.94	1.97	2.36	2.08	0.25%	−51.72%	−1.26%	8.33%
15*	3.99	1.26	2.71	2.8	10.53%	−80.06%	−13.69%	1.45%
16	2.45	5.07	2.9	2.31	5.60%	−31.76%	3.94%	0.87%
17	3.21	1.64	2.7	2.43	3.22%	−46.23%	−25.62%	−23.82%
18	2.44	7.3	2.66	1.75	−2.01%	14.78%	18.22%	−4.89%
19	1.92	31.8	5.04	2.83	0.52%	41.71%	17.21%	2.91%
20	3.6	7.59	3.55	2.63	−4.76%	2.43%	−3.79%	−7.72%
21	2.64	9.65	3.32	2.27	0.00%	2.66%	−17.21%	−21.18%
22	3.58	3.04	2.53	1.96	−9.37%	−45.23%	−13.36%	−9.68%
23	2.53	5.35	3.01	2.5	6.30%	−14.13%	−15.45%	−20.63%
24	2.46	10.18	4.1	2.82	−0.81%	−6.35%	6.22%	7.63%
25	2.69	8.68	3.7	2.36	−1.82%	−11.97%	1.09%	−2.07%
26	2.88	6.06	3.76	2.95	−2.70%	−20.89%	−5.29%	−3.59%
27	—	4.15	3.02	2.51	—	−13.18%	1.68%	4.58%
28	—	4.97	3.27	2.68	—	−7.45%	−12.33%	1.52%
29	1.74	13.77	3.52	2.3	8.75%	−2.06%	−3.56%	−9.45%
30	2.96	3.23	2.7	2.04	25.42%	−38.24%	3.85%	−0.49%
31	2.43	9.41	3.33	2.05	−3.95%	−0.53%	16.84%	5.67%
32	2.95	9.42	4.11	2.81	−1.99%	−54.49%	−14.91%	−14.33%
33	—	6.29	4.17	3.86	—	−58.94%	−15.07%	−1.03%
34	—	10.36	2.97	2.25	—	−48.12%	−29.29%	−21.05%
35	—	11.2	4.02	2.55	—	−31.37%	−9.66%	−13.56%
36	—	6.8	2.32	1.69	—	19.72%	9.95%	−0.59%
37	—	12.02	3.39	2.4	—	−43.28%	−22.78%	−16.08%
38	2.81	3.15	2.51	1.96	2.93%	−47.93%	−18.24%	−15.88%
39	1.69	10.27	2.94	1.86	−6.63%	−34.75%	−22.63%	−22.50%

				Mean	0.04	−0.21	−0.09	−0.08
				Number	31	39	39	39
				Test statistic	1.6	−4.41	−4.49	−5.41
				Critical value of *t*.05, *n*−1				
				1 tailed	2.04	2.02	2.02	2.02
				2 tailed	1.7	1.68	1.66	1.64
				*P* value	0.10 > *x* > 0.05	*x* < 0.005	*x* < 0.005	*x* < 0.005

*denotes patient referenced in Figure 1.
